# Assessment of Perceived Facial Age Changes Following Orthognathic Surgery Using Artificial Intelligence

**DOI:** 10.3390/healthcare14142200

**Published:** 2026-07-21

**Authors:** Özlem Elverişli, Hilal Alan, Ümit Yolcu, Ayşegül Evren

**Affiliations:** 1 Fulya Oral and Dental Health Polyclinic, Tekirdag 59000, Türkiye; 2 Private Malatya Academy Oral and Dental Health Polyclinic, Malatya 44000, Türkiye; hilal.alan@inonu.edu.tr; 3 Department of Oral and Maxillofacial Surgery, Faculty of Dentistry, Istanbul Medeniyet University, Istanbul 34000, Türkiye; umit.yolcu@medeniyet.edu.tr; 4 Etimesgut Oral and Dental Health Center, Ankara 06000, Türkiye; aysegul.evren@inonu.edu.tr

**Keywords:** artificial intelligence, orthognathic surgery, perceived age, facial esthetics, flourishing, social appearance anxiety

## Abstract

**Highlights:**

**What are the main findings?**

- AI-estimated facial age showed no significant change following orthognathic surgery (mean difference [preoperative−postoperative] 0.11 years, 95% CI −0.87 to 1.09; Cohen’s dz = 0.04), and the confidence interval excluded the previously reported 1.31-year reduction.
- Social appearance anxiety was lower and self-rated flourishing higher after surgery than before, based on questionnaires completed retrospectively for the pre-surgery state and concurrently for the post-surgery state.

**What are the implications of the main findings?**

- Improvements in psychosocial measures were observed following orthognathic surgery even without a statistically significant, AI-detected reduction in facial age; because the design was uncontrolled and the psychosocial ratings were retrospective, these associations cannot be interpreted as caused by surgery alone.
- AI-based facial analysis may complement patient-reported outcome measures in evaluating orthognathic surgery outcomes, but validated, reliability-tested tools are needed before AI-estimated age can serve as a primary outcome measure.

**Abstract:**

Background/Objectives: Orthognathic surgery is performed to improve facial esthetics and function in patients with dentofacial deformities. This study aimed to evaluate the impact of orthognathic surgery on perceived facial age using an artificial intelligence (AI)-based age-estimation tool and to examine accompanying changes in self-rated flourishing and social appearance anxiety. Methods: This retrospective, single-arm, before-and-after study included 27 patients who underwent bilateral sagittal split ramus osteotomy (BSSRO) and/or Le Fort I osteotomy. Preoperative and postoperative standardized frontal photographs were analyzed with the AI-based application “AgeBot: How Old Do I Look?”. Participants also completed the Social Appearance Anxiety Scale (SAAS) and the Flourishing Scale (FS; Turkish adaptation by Telef). Both questionnaires were completed at a single postoperative interview—first for the remembered preoperative state and then for the current postoperative state—so the psychosocial pre–post comparisons reflect retrospectively perceived change. Normality was assessed on the paired differences; the primary AI-age outcome was analyzed with the paired *t*-test (confirmed by the Wilcoxon signed-rank test) and the psychosocial outcomes with the Wilcoxon signed-rank test, with effect sizes reported. A data-informed sensitivity analysis, rather than an a priori power calculation, accompanied the primary outcome. Results: AI-estimated facial age did not change significantly after surgery (mean difference [preoperative−postoperative] 0.11 ± 2.49 years, 95% CI −0.87 to 1.09, *p* = 0.818; Cohen’s dz = 0.04), and the confidence interval excluded the previously reported 1.31-year reduction. Exploratory change-score comparisons showed no significant differences by sex or smoking status. Postoperative SAAS scores were lower than retrospective preoperative scores (median 37 [IQR 24.5–42.0] vs. 51 [43.0–56.0]; Z = −4.07, *p* < 0.001, r = 0.55), and postoperative FS scores were higher (median 46 [40.0–51.5] vs. 45 [38.5–45.0]; Z = −2.45, *p* = 0.014, r = 0.37). Conclusions: Orthognathic surgery was not associated with a statistically significant change in AI-estimated facial age in this small cohort. More favorable social appearance anxiety and flourishing scores were observed following treatment, but the retrospective, uncontrolled assessment precludes causal attribution. AI-assisted facial age estimation may complement patient-reported outcomes, but validated, repeatability-tested tools are required before it can serve as a primary outcome measure.

## 1. Introduction

Orthognathic surgical treatment is a treatment method that corrects functional and esthetic disorders of dentofacial deformities through the cooperation of orthodontics and surgery to create more harmonious facial and skeletal relationships [1,2]. Patients apply to clinics due to dissatisfaction with facial appearance, psychosocial problems, or functional disorders related to speech or breathing [2]. Following aesthetic facial surgeries, patients report a significant increase in satisfaction with their appearance and quality of life. They also report positive physical, emotional, and social effects [3].

Facial appearance plays an important role in psychosocial well-being and quality of life. Previous studies have shown that correction of dentofacial deformities through orthognathic surgery may lead to improvements in body image, self-confidence, and social functioning [4,5,6,7]. Enhanced facial harmony following treatment has also been associated with better self-esteem and greater satisfaction with social interactions [8].

Recent advances in artificial intelligence (AI) have expanded its applications in medical image analysis and facial recognition. Although facial attractiveness remains a subjective concept, AI-based systems can analyze facial characteristics from photographs and estimate apparent age [1,9]. In orthognathic surgery, AI technologies are increasingly used for image processing, treatment planning, surgical customization, and outcome assessment [9]. These developments suggest that AI may offer valuable tools for a more rater-independent evaluation of facial changes following treatment.

Reproducible, quantifiable assessment of age-related facial change is clinically important because conventional evaluation methods—panel ratings by orthodontists, surgeons, or laypeople using photographs, silhouettes, or line drawings—are vulnerable to observer bias, panel-composition effects, and limited inter-study reproducibility [10,11,12,13,14,15]. AI-based age-estimation tools remove this rater-dependency and have already been used to quantify esthetic outcomes in other cosmetic and reconstructive procedures. Dorfman et al. used a deep-learning age-estimation algorithm to show that patients appeared approximately three years younger after open rhinoplasty than before surgery [16], and Zimm et al. used structured observer-based apparent-age assessment to document perceived age reversal after aging-face (facelift) surgery [17]. These precedents from other facial cosmetic procedures provided the methodological rationale for applying a comparable AI-based approach to orthognathic surgery, an area in which the effect of treatment on perceived age has not previously been examined using an automated, rater-independent tool.

Despite increasing evidence regarding the esthetic and psychosocial benefits of orthognathic surgery, its influence on perceived facial age remains unclear. Artificial intelligence-based facial analysis systems may provide rater-independent approach for evaluating age perception while minimizing observer-related bias. Given the growing integration of AI technologies into healthcare and facial image analysis, assessing age-related changes after orthognathic surgery may provide additional insight into treatment outcomes. Therefore, this study investigated AI-estimated facial age before and after orthognathic surgery and explored whether potential changes were accompanied by differences in self-rated flourishing and social appearance anxiety. The study investigated whether orthognathic surgery was associated with changes in AI-estimated facial age and psychosocial outcomes.

## 2. Material and Methods

### 2.1. Ethical Approval

This study was approved by the Inonu University Health Sciences Non-Interventional Clinical Research Ethics Committee with the number 2022/3735 on 20 September 2022.

Because standardized facial photographs were analyzed using a commercial AI-based age-estimation application, the handling of these images is described explicitly. The institutional ethics application specified that standardized facial photographs would be evaluated using an AI-based age-analysis program, and all participants provided written informed consent for the use of their facial photographs for research purposes. No accompanying identifiers (patient names, identification numbers, or medical-record data) were transmitted with the images; however, because a recognizable facial photograph is itself potentially identifying, these images are more accurately described as identifier-stripped (pseudonymized) rather than fully de-identified or anonymized. This procedure was consistent with the approved ethics protocol and with the Declaration of Helsinki.

### 2.2. Study Design

This retrospective before-and-after (single-arm) study was conducted and reported in accordance with the Strengthening the Reporting of Observational Studies in Epidemiology (STROBE) guidelines.

**Figure 1:**
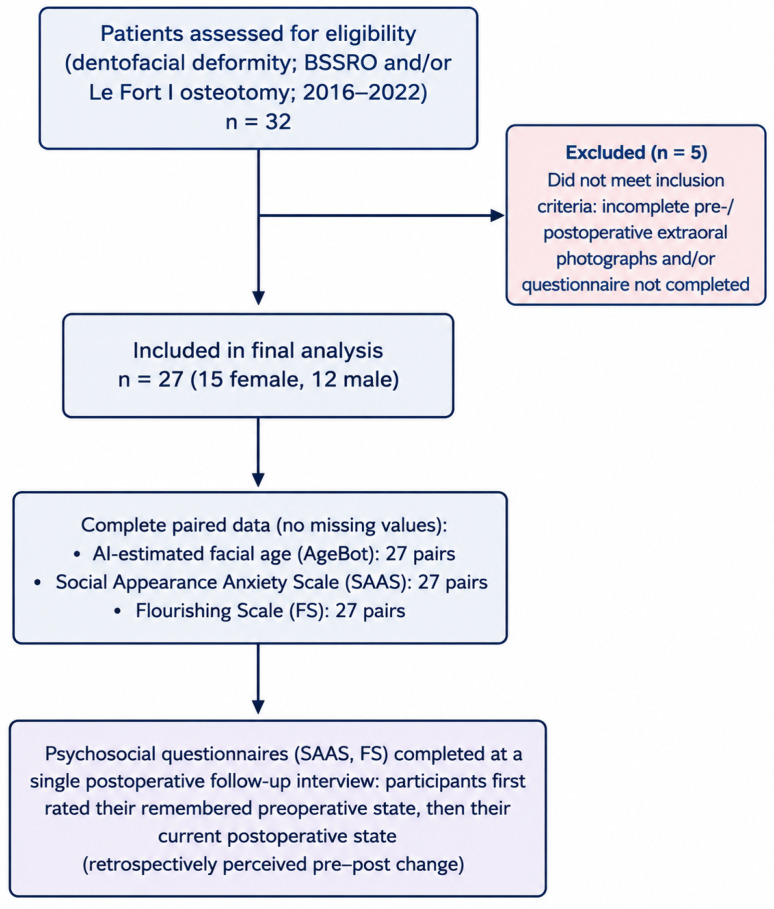
Participant flow diagram. Preoperative psychosocial questionnaires (SAAS and Flourishing Scale) were completed retrospectively at the postoperative interview (Section 2.6).

In this study, 32 patients referred to Inönü University, Faculty of Dentistry, Department of Oral, Dental and Maxillofacial Surgery between 2016 and 2022 with a diagnosis of dentofacial deformity, who underwent bilateral sagittal split ramus osteotomy (BSSRO) or Le Fort I osteotomy under general anesthesia (or both procedures simultaneously), were assessed for eligibility. Of these, 5 were excluded because they did not meet the inclusion criteria (incomplete preoperative or postoperative extraoral photographs and/or non-completion of the questionnaire), leaving 27 patients (15 female, 12 male) in the final analysis. All 27 analyzed participants had complete paired photographic and questionnaire data, with no missing values; the participant flow is summarized in Figure 1.

Patients between the ages of 18–40 years, who underwent BSSRO or LeFort I osteotomy for correction of Class II or Class III maxillofacial deformity, who had completed skeletal growth and development, who had complete photographic and radiographic records, who had no systemic or metabolic disease, and who had no history of trauma to the maxillofacial region were included in the study.

Patients whose growth and development were incomplete, who had any syndrome involving the craniofacial region, who had incomplete preoperative and postoperative extraoral photographs, and who did not participate in the questionnaire study were excluded.

All of the individuals included in the study underwent orthodontic treatment before orthognathic surgery. After dental corrections were completed, a surgical arch was made and surgical splints were prepared to bring the maxilla and mandible to their planned positions during surgery. The patients included in the study underwent nasal intubation under general anesthesia and bilateral sagittal split ramus osteotomy and/or LeFort I osteotomy by the same surgical team.

### 2.3. Sample Size

Because of the limited number of eligible patients treated during the study period, all consecutive patients meeting the inclusion criteria were recruited. Sample size determination and statistical power analysis were performed for the primary outcome.

### 2.4. Power Analysis

A target effect size for the primary outcome (the change in perceived facial age following orthognathic surgery) was derived from the study by Pourtaheri et al. [18], which reported a mean reduction of 1.31 years in perceived facial age after orthognathic surgery; this value was taken as the anticipated clinically relevant difference.

Because Pourtaheri et al. [18] did not report the standard deviation of paired differences, the standard deviation observed in the present dataset was used to estimate a standardized effect size (Cohen’s dz = 0.53). Consequently, this should be regarded as a data-informed sensitivity/achieved-power estimate rather than a conventional prospective a priori power analysis. Accordingly, the resulting calculation should be interpreted as this makes the resulting calculation a data-informed sensitivity/achieved-power estimate rather than a strictly independent, purely prospective a priori calculation, since the variance component was not sourced from external literature. This calculation is therefore presented as a methodological limitation (see Section 4.2) rather than presenting the analysis as a conventional a priori power calculation. For a paired-samples *t*-test with a two-sided alpha level of 0.05 and 80% statistical power, the minimum required sample size using this effect size was calculated as 31 patients.

However, due to the limited number of eligible patients treated during the study period, a total of 27 patients were included in the final analysis. The final years of the recruitment period overlapped with the COVID-19 pandemic, during which elective oral and maxillofacial surgical procedures, including orthognathic surgery, were substantially reduced or postponed [19]. Therefore, the target sample size of 31 patients could not be reached, and the study was completed with 27 patients. With the final sample size (*n* = 27), the estimated statistical power was approximately 74.9%.

Accordingly, this calculation should be interpreted as a data-informed sensitivity analysis rather than a conventional a priori power calculation (see Section 4.2). Interpretation of the primary outcome therefore rests principally on the observed effect size and its 95% confidence interval: the mean difference of 0.11 years (95% CI −0.87 to 1.09) was small, and its confidence interval excluded the 1.31-year reduction anticipated from Pourtaheri et al. [18], indicating no detectable reduction in AI-estimated facial age rather than an uninformative result.

### 2.5. Artificial Intelligence Application

AgeBot (“AgeBot: How Old Do I Look?”, developed by RoboBot Studio) is a commercially available, artificial intelligence-based facial age-estimation application that analyzes facial features from a digital photograph and returns an estimated age. It was used to obtain an AI-assisted age estimate rather than an independently validated or “objective” measurement (see Section 4.2). All photographs were acquired and analyzed under standardized conditions, and the same application and upload procedure were used for every participant. Because the estimated age was generated automatically by the application, no manual scoring or adjustment by the investigator was involved. However, the image analysis was not performed under formal blinding to time point, and several operational details relevant to reproducibility—including the specific application build, the operating system and device used for analysis, the exact dates of analysis, and the stored image resolution—were not systematically recorded. These limitations, together with the absence of published validation for AgeBot, are discussed in Section 4.2.

**Figure 2:**
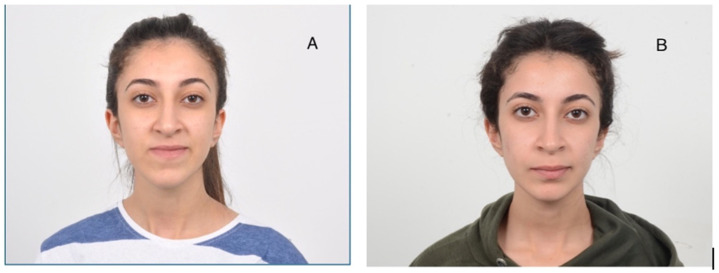
Standardized facial photographs before (**A**) and after (**B**) orthognathic surgery.

All 27 patients included in the study underwent extraoral frontal photography at both the preoperative and 6-month postoperative time points. Photographs were obtained by the same experienced technician using the same camera (Nikon D90, Nikon Corporation, Tokyo, Japan) under standardized conditions, with the subjects in the natural head position and their lips closed without tension. Background, ambient lighting, and camera-to-subject distance were held constant across all photographic sessions to minimize non-facial sources of variation in the AI age estimate (Figure 2).

Each photograph was submitted to AgeBot as a single analysis pass, and no repeat-run (test–retest) reliability assessment of the application was performed in this study; the absence of a validated repeatability metric for AgeBot is acknowledged as a limitation in Section 4.2, and small numerical differences in estimated age (on the order of the reported mean difference) should accordingly be interpreted with caution, as they cannot be distinguished from measurement noise inherent to an unvalidated instrument.

Photographs were analyzed as full frontal images without cropping to isolate the facial region; consequently, non-facial cues potentially present in the frame (e.g., hairstyle or clothing visible at the image periphery) cannot be fully excluded as a source of variability in the AI-estimated age, despite the standardization of background, lighting, and camera distance described above. Future studies should consider cropping images to the facial region alone before AI analysis to remove this potential confound.

To date, AgeBot has not been the subject of a published, peer-reviewed validation study establishing its accuracy or test–retest reliability, in contrast to validated convolutional neural network (CNN)-based age-estimation algorithms (e.g., the DEX/Rothe et al. architecture used by Patcas et al. [1]) that have been benchmarked against known chronological ages in large training datasets. AgeBot was selected for this study primarily because of its consumer popularity (download volume and user ratings) rather than documented scientific validation; this choice, and its implications for the interpretation of the primary outcome, is discussed further in Section 4.

**Figure 3:**
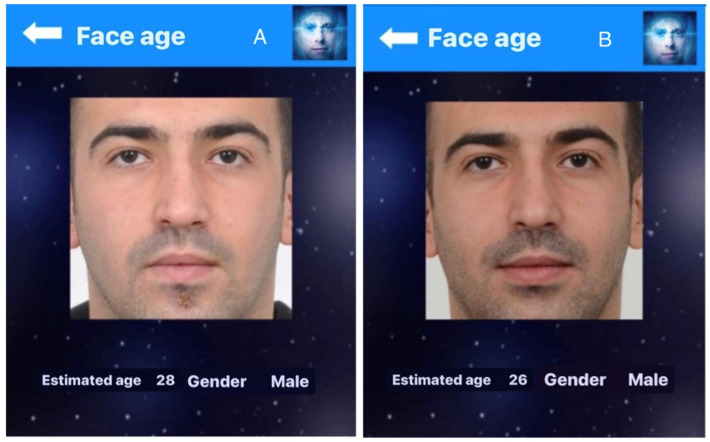
AI-based facial age assessment before (**A**) and after (**B**) surgery.

The preoperative and postoperative frontal photographs of the patients were evaluated with the artificial intelligence-based “AgeBot: How old do I look?” application developed by RoboBot Studio (Figure 3).

### 2.6. Survey Application

A questionnaire comprising three sections was administered. The first section was a demographic form covering age, sex, and smoking. The second section was the Social Appearance Anxiety Scale (SAAS), which measures anxiety arising from one’s physical appearance [20,21]. The SAAS is a 16-item, single-factor, 5-point Likert scale (total score range 16–80, with higher scores indicating greater social appearance anxiety) developed by Hart et al. [20]; the validated Turkish adaptation (Cronbach’s α = 0.93, test–retest reliability = 0.85) was reported by Doğan [21]. The third section was the Flourishing Scale (FS) developed by Diener et al. [22], an 8-item, unidimensional measure of self-perceived success and psychological flourishing (e.g., purpose, positive relationships, engagement, and optimism). Each item is rated on a 7-point Likert scale from 1 (strongly disagree) to 7 (strongly agree), and the items are summed to a single total score ranging from 8 to 56, with higher scores indicating greater self-rated flourishing; the instrument yields one overall score and does not produce subscale dimensions. The validated Turkish adaptation, for which a Cronbach’s α of 0.80 was reported, was published by Telef [23]. No standardized clinical cut-off thresholds are defined for either instrument; scores are interpreted continuously, with higher scores reflecting greater social appearance anxiety (SAAS) or greater flourishing (FS). Both questionnaires were administered at a single postoperative follow-up interview: participants first completed each questionnaire retrospectively, reporting how they recalled their preoperative status, and then completed the same questionnaires for their current postoperative status.

### 2.7. Statistical Method

Statistical analyses were performed in SPSS version 22.0 (IBM SPSS Statistics, Chicago, IL, USA); all procedures used (Shapiro–Wilk test, paired *t*-test, Wilcoxon signed-rank test, and one-way ANCOVA) are standard methods whose numerical results do not depend on this software version. In keeping with the paired design, the normality assumption was evaluated on the within-participant paired differences (rather than on the baseline scores) using the Shapiro–Wilk test. For the primary outcome (AI-estimated age), the paired differences showed only mild departure from normality (W = 0.92, *p* = 0.041). Because the paired *t*-test is generally robust to mild departures from normality at this sample size, it was retained as the primary analysis. A Wilcoxon signed-rank test was additionally performed as a confirmatory sensitivity analysis (*p* = 0.517). For the psychosocial outcomes, the paired differences were non-normal for the Flourishing Scale (W = 0.82, *p* < 0.001) and approximately normal for the SAAS (W = 0.96, *p* = 0.29); the Wilcoxon signed-rank test was applied to both to provide a consistent, conservative treatment of these ordinal-derived totals, and a paired *t*-test for the SAAS gave the same conclusion (*p* < 0.001). Continuous outcomes are summarized as mean ± SD and, for the non-parametrically analyzed measures, as median (interquartile range, IQR). Effect sizes are reported as Cohen’s dz for the paired *t*-test and as the rank-based r (r = Z/√N, where N is the number of observations) together with the matched-pairs rank-biserial correlation for the Wilcoxon tests, with Hodges–Lehmann median differences and bootstrap 95% confidence intervals for the psychosocial change. Differences in change scores between sex and smoking subgroups were compared using the independent-samples *t*-test and the Mann–Whitney U test; owing to small subgroup sizes, these subgroup analyses are exploratory. Statistical significance was set at *p* < 0.05.

The effect of smoking status on postoperative AI-estimated facial age was evaluated with a one-way analysis of covariance (ANCOVA): the dependent variable was postoperative AI-estimated facial age, the fixed factor was smoking status (smoker/non-smoker), and preoperative AI-estimated facial age was the covariate. The assumptions of ANCOVA were examined. Homogeneity of regression slopes was supported by a non-significant smoking × covariate interaction (F(1,23) = 0.14, *p* = 0.712). Homoscedasticity was confirmed using Levene’s test (*p* = 0.29). Residuals showed only mild deviation from normality (Shapiro–Wilk W = 0.91, *p* = 0.021), a condition under which ANCOVA is generally considered robust. Data are presented as mean ± SD, median (IQR), frequency (%), or number (%), as appropriate.

## 3. Results

### 3.1. General Characteristics of Patients

Twenty-seven patients completed the study protocol and were included in the final analysis. The mean age of participants was 22.51 ± 3.89 years. Of the participants, 55.6% were female and 44.4% were male. Eight participants (29.6%) reported active smoking, and 19 (70.4%) did not (Table 1).

### 3.2. Evaluation of Preoperative and Postoperative Estimated Ages, Including Exploratory Subgroup Analyses by Sex and Smoking Status

The mean preoperative estimated age was 23.74 ± 5.33 years and the mean postoperative estimated age was 23.62 ± 4.50 years, as determined by the age-analysis application. The change in AI-estimated facial age following orthognathic surgery was not statistically significant (mean difference [preoperative − postoperative] 0.11 ± 2.49 years, 95% CI −0.87 to 1.09, t(26) = 0.232, *p* = 0.818; Cohen’s dz = 0.04). The change in AI-estimated facial age following orthognathic surgery was not statistically significant (mean difference 0.11 ± 2.49 years, 95% CI −0.87 to 1.09, t(26) = 0.232, *p* = 0.818). A confirmatory Wilcoxon signed-rank test yielded the same conclusion (*p* = 0.517). The 95% confidence interval excluded the 1.31-year reduction reported by Pourtaheri et al. [18], indicating no detectable reduction in AI-estimated facial age (Table 2).

In exploratory analyses, the mean preoperative and postoperative estimated ages were 25.83 ± 5.27 and 25.08 ± 4.54 years in male patients (*n* = 12) and 22.07 ± 4.93 and 22.47 ± 4.27 years in female patients (*n* = 15). To assess whether the change in AI-estimated age differed by sex, the individual change scores were compared directly between groups; this difference was not statistically significant (male mean change 0.75 years vs. female −0.40 years; Welch t = −1.25, *p* = 0.222; Mann–Whitney U, *p* = 0.348).

Similarly, the mean preoperative and postoperative estimated ages were 24.75 ± 7.07 and 25.00 ± 5.58 years in smokers (*n* = 8) and 23.32 ± 4.59 and 23.05 ± 4.01 years in non-smokers (*n* = 19). When change scores were compared directly, the change in AI-estimated age did not differ significantly between smokers and non-smokers (smoker mean change −0.25 years vs. non-smoker 0.26 years; Welch t = 0.54, *p* = 0.597; Mann–Whitney U, *p* = 0.248). Because these subgroups are small (*n* = 8–15), all subgroup analyses are exploratory; the within-group paired tests in Table 2 are presented for description only and were not used to infer between-group differences.

Because these sex- and smoking-status subgroups are small (*n* = 8–15) and subgroup-level normality could not be independently confirmed (Section 2.7), these comparisons should be regarded as exploratory rather than confirmatory.

A one-way ANCOVA, with postoperative AI-estimated facial age as the dependent variable, smoking status as the fixed factor, and preoperative AI-estimated facial age as the covariate, showed that smoking status was not significantly associated with postoperative AI-estimated facial age after adjustment for baseline age (F(1,24) = 0.961, *p* = 0.337, partial η^2^ = 0.039). Preoperative AI-estimated facial age was a significant covariate (F(1,24) = 87.392, *p* < 0.001, partial η^2^ = 0.785), and the model explained 79.3% of the variance in postoperative AI-estimated facial age (R^2^ = 0.793, adjusted R^2^ = 0.776). The ANCOVA assumptions were satisfied: homogeneity of regression slopes (smoking × covariate interaction F(1,23) = 0.14, *p* = 0.712) and homoscedasticity of residuals (Levene’s test, *p* = 0.29) were met, and the residuals showed only mild, non-influential deviation from normality (Shapiro–Wilk W = 0.91, *p* = 0.021) (Table 3).

Retrospectively assessed social appearance anxiety was more favorable after surgery: the postoperative SAAS score (median 37, IQR 24.5–42.0; mean 35.41 ± 11.64) was lower than the retrospective preoperative score (median 51, IQR 43.0–56.0; mean 48.11 ± 12.41), a statistically significant difference (Z = −4.07, *p* < 0.001; r = 0.55; matched-pairs rank-biserial = −0.90; Hodges–Lehmann median difference −12.5, 95% CI −16.0 to −4.0). Self-rated flourishing was higher after surgery: the postoperative Flourishing Scale score (median 46, IQR 40.0–51.5; mean 45.93 ± 7.82) exceeded the retrospective preoperative score (median 45, IQR 38.5–45.0; mean 42.11 ± 8.62), also a statistically significant difference (Z = −2.45, *p* = 0.014; r = 0.37; matched-pairs rank-biserial = 0.60; Hodges–Lehmann median difference 3.5, 95% CI 0.0 to 6.0) (Table 4). Because the preoperative scores were recalled retrospectively at the same interview as the postoperative scores, these differences represent retrospectively perceived change and are interpreted with corresponding caution (see Section 4.2).

## 4. Discussion

The principal finding of this study was that orthognathic surgery was not associated with a statistically significant change in AI-estimated facial age, whereas retrospectively assessed social appearance anxiety and self-rated flourishing were more favorable after surgery. For the primary outcome, the small mean difference (0.11 years) and its 95% confidence interval (−0.87 to 1.09), which excluded the 1.31-year reduction anticipated from earlier work [18], indicate no detectable effect on AI-estimated perceived age; this interpretation is supported by the accompanying data-informed sensitivity analysis (Section 2.4). These findings indicate that any detectable impact of orthognathic surgery on AI-estimated perceived age may be limited in young adult populations [17,24,25], even though the psychosocial measures moved in a favorable direction.

Previous studies evaluating the esthetic outcomes of orthognathic treatment have generally reported improved attractiveness ratings by orthodontists, surgeons, and laypeople using silhouettes, line drawings, and photographs [10,11,12,13,14,15,17], and an esthetically flat facial profile has been linked to a perceived age benefit in patients with dentofacial deformity [18,26,27]. Using a CNN-based algorithm, Patcas et al. found that patients with dentofacial deformity appeared older than their chronological age and that this gap narrowed after treatment, particularly in women and in patients undergoing bimaxillary surgery [1]. Pourtaheri et al. similarly reported a reduction in perceived age after orthognathic surgery, most pronounced in older patients and in those with concave profiles [18]. In contrast, and consistent with our findings, Siu found no significant change in perceived age despite improved attractiveness ratings [24], and Denadai et al. reported improved attractiveness but no change in perceived facial age using FACE-Q assessment [28]. Dorfman et al., using an AI age-estimation algorithm in rhinoplasty patients, found that patients appeared roughly three years younger postoperatively [16].

Notably, the studies reporting no change in perceived age despite improved attractiveness (Siu [24]; Denadai et al. [28]) relied on human observers rather than an automated tool, broadly consistent with our AI-based finding. Because this study did not include a parallel human-rater arm, we cannot determine whether AI-estimated age changes track how human observers perceive these patients; we recommend that future studies pair AI-based estimation with a masked human-rater panel assessing the same photographs.

CNN-based age-estimation algorithms have been shown to outperform human raters [16], but unlike these validated architectures [1,16], AgeBot has not undergone peer-reviewed validation, and no test–retest reliability or comparison against a second estimator was performed here (Section 2.5); the small mean difference observed (0.11–0.12 years) could plausibly reflect measurement noise rather than a true effect. A possible biological explanation for the null result is that younger patients typically show fewer textural signs of skin aging, so soft-tissue changes following skeletal correction may be less perceptible in this predominantly young cohort than in older patients [26,27]; a true modest effect cannot be fully excluded, given the width of the confidence interval reported in Section 2.4. AgeBot was selected primarily for its consumer popularity rather than documented scientific validation [1], and although using the same application at both time points removes between-instrument variability, it does not establish within-instrument reliability—both are acknowledged as limitations of the present design.

Multi-planar photography (frontal, oblique, lateral, and basal) is generally recommended for evaluating orthognathic outcomes, as lateral and oblique views better capture the profile changes surgery is intended to produce; however, AgeBot and similar consumer tools cannot analyze lateral images, which constrains our frontal-only findings. Smoking has been associated with accelerated skin aging via microvascular and connective-tissue effects [29,30], but we found no significant difference in predicted facial age between smokers and non-smokers; with only 8 smokers in this cohort, this comparison is markedly underpowered and should be regarded as preliminary rather than a reliable estimate of the true effect of smoking status. Postoperative assessment was performed at 6 months; this interval was selected pragmatically, to balance adequate healing time against the follow-up window feasible within a retrospective single-center cohort, rather than being derived from a specific evidence-based optimal interval established for AI-based age assessment. Because facial soft-tissue edema after orthognathic surgery can persist at a residual level of roughly 10% at this time point [31], with further resolution up to 12 months, some rejuvenation effect may not yet have been fully apparent.

Improvements in facial appearance after orthognathic surgery are consistently associated with better psychosocial adjustment and quality of life [4,6,7,8,32,33,34,35,36]; for example, Kovalenko et al. linked greater deformity severity to higher emotional instability and anxiety [7], and Øland et al. found that an improved facial profile positively affected self-esteem and social life [8]. In our study, the more favorable postoperative SAAS and Flourishing Scale scores are consistent with this literature; however, because the psychosocial questionnaires were completed retrospectively at a single postoperative interview and the design lacked a concurrent control group, these changes cannot be attributed specifically to surgery and may partly reflect recall bias, response shift, expectancy effects, or regression to the mean (Section 4.2). Exploratory comparisons of individual change scores showed no significant differences by sex or smoking status (all *p* > 0.12); age and type of skeletal deformity were not examined as moderators, and no claim is made regarding these factors.

### 4.1. Clinical Implications

Taken together, these findings suggest that clinicians should not promise patients a measurable, AI-detectable reduction in perceived facial age. Participants reported more favorable social appearance anxiety and flourishing scores after surgery; however, because these were the only psychosocial constructs measured and were assessed retrospectively without a control group, they should be described as improvements observed following surgery in the constructs actually measured, rather than as demonstrated gains in self-confidence or social functioning or as evidence of facial rejuvenation.

### 4.2. Limitations

This study has several strengths, including the use of standardized photographic records, evaluation by a single AI-based assessment system, and simultaneous assessment of psychosocial outcomes. Nevertheless, several limitations should be acknowledged.

First, the study enrolled 27 of the 31 patients indicated by the sample-size calculation. Because the variance used to derive the target effect size (Cohen’s dz = 0.53) was estimated from the study data, this calculation should be interpreted as a data-informed sensitivity analysis rather than an independent a priori power analysis. Accordingly, interpretation of the primary outcome is based primarily on the observed effect size and its 95% confidence interval rather than on achieved statistical power.

Second, psychological flourishing was assessed using the Flourishing Scale [22], an 8-item unidimensional instrument. Because the original item-level questionnaire responses were no longer available, internal consistency (Cronbach’s α) could not be recalculated for the present sample. Therefore, we relied on the published psychometric properties of the validated Turkish version of the scale [23].

Third, an important limitation of the psychosocial outcomes is that the SAAS and Flourishing Scale questionnaires were not administered prospectively before surgery. Participants were contacted at postoperative follow-up and, in a single interview, first completed each questionnaire for their remembered preoperative state and then for their current postoperative state. The preoperative psychosocial scores are therefore retrospectively recalled ratings, and the pre–post psychosocial comparisons are best understood as retrospectively perceived change. This procedure is susceptible to recall bias, to response-shift bias (a change in the respondent’s internal standards or conceptualization of the construct after treatment), and to expectancy effects, each of which could inflate the apparent pre–post difference. These psychosocial results should accordingly be interpreted with caution and not as prospectively measured change.

Fourth, AI-estimated facial age was compared as a raw preoperative-versus-postoperative value and was not adjusted for the participants’ true chronological aging between the two photographs (approximately six months). An analysis expressing the outcome as AI-estimated age minus chronological age at the date of each photograph could not be performed because the exact dates of the standardized photographs were unavailable in this retrospective cohort. Therefore, the findings should be interpreted in light of this limitation.

Fifth, the age-estimation instrument itself has important limitations. AgeBot has no published, peer-reviewed validation of its accuracy or repeatability; each photograph was analyzed in a single pass, so no test–retest reliability was established, and the output was not compared against a second AI tool, a validated CNN-based algorithm, or a masked human-rater panel. Images were analyzed as full frontal photographs without cropping to the facial region, so peripheral non-facial cues (e.g., hairstyle or clothing) cannot be fully excluded as sources of variability, and only frontal views were assessed because consumer tools of this type cannot analyze lateral or oblique images. Several operational details relevant to reproducibility were not recorded (Section 2.5). These limitations, together with the absence of published validation for AgeBot, mean that the results should be interpreted as AI-assisted estimates rather than objective measurements.

Postoperative assessment was performed at 6 months, chosen pragmatically to balance healing time against the follow-up feasible in a retrospective cohort rather than a literature-derived optimum; because roughly 10% of postoperative facial soft-tissue edema may persist at this point, with further resolution up to 12 months [31], any rejuvenation effect might not yet have been fully apparent. In addition, this single-arm, before-and-after design has no concurrent control group, so even the AI-based comparison cannot isolate a specific surgical effect from non-specific changes over time, and the smoking subgroup (*n* = 8) is too small to support confident conclusions; subgroup analyses by sex and smoking status should be regarded as exploratory.

The overall sample was small and predominantly composed of young adults, and only frontal photographs were analyzed; because younger patients show fewer textural signs of skin aging, the findings may not generalize to older patients. With respect to data handling, although direct textual identifiers were removed, facial photographs remain potentially identifiable biometric data. Their use was covered by the institutional ethics committee and covered by written informed consent for research purposes (Section 2.1). Future studies should use validated AI age-estimation systems with documented test–retest reliability, prospectively collected psychosocial measures, adequately powered and more age-diverse samples, human-rater comparison arms, and appropriate control groups.

## 5. Conclusions

Orthognathic surgery was not associated with a statistically significant change in AI-estimated facial age in this small cohort of predominantly young adults; the mean difference was 0.11 years (95% CI −0.87 to 1.09), a confidence interval that excluded the previously reported 1.31-year reduction. More favorable social appearance anxiety and self-rated flourishing scores were observed following treatment, although these psychosocial measures were assessed retrospectively and without a control group and therefore cannot be attributed specifically to surgery. Because no parallel human-rater arm was included, the premise that AI-based facial analysis provides a more rater-independent alternative to human perception of age—reflected in the title of this article—remains an assumption rather than a demonstrated finding and should be tested directly in future work comparing AI-estimated and human-perceived age on the same photographs. Adequately powered, controlled studies using externally sourced effect-size estimates, validated and repeatability-tested AI tools, prospectively collected psychosocial outcomes, and follow-up beyond 6 months are needed to clarify these associations.

## Figures and Tables

**Table 1:** Demographic and clinical characteristics of the study participants.

		*n* = 27 (Mean ± SD)	Frequency (%)
**Age (year)**			22.51 ± 3.89
Sex (Female/Male)	Female	15	55.6
	Male	12	44.4
Smoking cigarettes (Yes/No)	Yes	8	29.6
	No	19	70.4

Values are presented as mean ± standard deviation (SD) or n (%), as appropriate.

**Table 2:** Preoperative and postoperative AI-estimated facial age with exploratory subgroup analyses according to sex and smoking status.

		Mean ± SD	Mean Difference (Preoperative − Postoperative)				
			Mean ± SD	95% CI	t	df	*p* *
Preop estimated age		23.74 ± 5.33	0.11 ± 2.49	−0.87 to 1.09	0.232	26	0.818
Postop estimated age		23.62 ± 4.50					
Male	Preop estimated age	25.83 ± 5.27	0.75 ± 1.88	−0.40 to 1.99	1.326	11	0.212
	Postop estimated age	25.08 ± 4.54					
Female	Preop estimated age	22.07 ± 4.93	−0.40 ± 2.80	−1.95 to 1.15	−0.554	14	0.589
	Postop estimated age	22.47 ± 4.27					
Smokers	Preop estimated age	24.75 ± 7.07	−0.25 ± 2.05	−1.97 to 1.47	−0.344	7	0.741
	Postop estimated age	25.00 ± 5.58					
Non-smokers	Preop estimated age	23.32 ± 4.59	0.27 ± 2.68	−1.03 to 1.56	0.427	18	0.674
	Postop estimated age	23.05 ± 4.01					

* Values are presented as mean ± standard deviation. Mean difference is calculated as preoperative minus postoperative AI-estimated facial age; a positive value indicates apparent age reduction after surgery. CI, confidence interval; df, degrees of freedom.

**Table 3:** Analysis of covariance (ANCOVA) evaluating the effect of smoking status on postoperative AI-estimated facial age after adjustment for preoperative AI-estimated facial age.

	Sum of Squares	df	Mean Square	F	*p*	Partial η^2^
Preoperative AI-estimated age (covariate)	397.723	1	397.723	87.392	<0.001	0.785
Smoking status	4.374	1	4.374	0.961	0.337	0.039
Error	109.225	24	4.551			
Corrected Total	528.296	26				

Abbreviations: ANCOVA, analysis of covariance. Dependent variable: postoperative AI-estimated facial age. Covariate: preoperative AI-estimated facial age. Overall model: R^2^ = 0.793 (adjusted R^2^ = 0.776).

**Table 4:** Changes in social appearance anxiety (SAAS) and self-rated flourishing (Flourishing Scale, FS) following orthognathic surgery.

	Mean ± SD	Median (IQR)	Wilcoxon *p* Value (Effect Size r)
Preop SAAS Score	48.11 ± 12.41	51 (43.0–56.0)	<0.001 (r = 0.55)
Postop SAAS Score	35.41 ± 11.64	37 (24.5–42.0)	
Preop FS Score	42.11 ± 8.62	45 (38.5–45.0)	0.014 (r = 0.37)
Postop FS Score	45.93 ± 7.82	46 (40.0–51.5)	

Values are presented as median (interquartile range, IQR) and mean ± standard deviation; the final column gives the Wilcoxon signed-rank *p* value and the rank-based effect size r (Z/√N). Observed score ranges were: SAAS 24–67 (preoperative) and 20–60 (postoperative); FS 16–56 (preoperative) and 26–56 (postoperative). Preoperative psychosocial scores were completed retrospectively at the postoperative interview (Section 2.6). SD, standard deviation; IQR, interquartile range; SAAS, Social Appearance Anxiety Scale; FS, Flourishing Scale.

## Data Availability

The data are not publicly available due to privacy and ethical restrictions but are available from the corresponding author upon reasonable request.
